# Single-cell RNA sequencing of freshly isolated bovine milk cells and cultured primary mammary epithelial cells

**DOI:** 10.1038/s41597-021-00972-1

**Published:** 2021-07-15

**Authors:** Doreen Becker, Rosemarie Weikard, Frieder Hadlich, Christa Kühn

**Affiliations:** 1 Leibniz Institute of Farm Animal Biology (FBN), Institute of Genome Biology, Wilhelm-Stahl-Allee 2, 18196 Dummerstorf, Germany; 2 University of Rostock, Faculty of Agricultural and Environmental Sciences, Justus-von-Liebig-Weg 6, 18059 Rostock, Germany

**Keywords:** Transcriptomics, Transcriptomics

## Abstract

Bovine mammary function at molecular level is often studied using mammary tissue or primary bovine mammary epithelial cells (pbMECs). However, bulk tissue and primary cells are heterogeneous with respect to cell populations, adding further transcriptional variation in addition to genetic background. Thus, understanding of the variation in gene expression profiles of cell populations and their effect on function are limited. To investigate the mononuclear cell composition in bovine milk, we analyzed a single-cell suspension from a milk sample. Additionally, we harvested cultured pbMECs to characterize gene expression in a homogeneous cell population. Using the Drop-seq technology, we generated single-cell RNA datasets of somatic milk cells and pbMECs. The final datasets after quality control filtering contained 7,119 and 10,549 cells, respectively. The pbMECs formed 14 indefinite clusters displaying intrapopulation heterogeneity, whereas the milk cells formed 14 more distinct clusters. Our datasets constitute a molecular cell atlas that provides a basis for future studies of milk cell composition and gene expression, and could serve as reference datasets for milk cell analysis.

## Abstract (summary)

**Table:** 

Measurement(s)	single-cell gene expression analysis
Technology Type(s)	10 × 3′ v3 • RNA sequencing
Sample Characteristic - Organism	Bos taurus
Sample Characteristic - Environment	mammary gland fluid/secretion • epithelium of mammary gland

Machine-accessible metadata file describing the reported data: 10.6084/m9.figshare.14611878

## Background & Summary

Bovine mammary structure, function, development and immune response and the underlying transcriptional regulatory processes in the mammary tissue have been studied extensively for almost half a century. Due to technical advances and new research techniques, molecular mechanisms of lactation, development and immune response can be analysed more accurately^1,2^. However, bovine mammary gland tissue can only be collected by biopsy or after slaughter, and comprises a number of heterogeneous cell types (e.g., mammary epithelial cells (MECs), myoepithelial cells, adipocytes, fibroblasts) creating potential bias in the outcome of global transcriptome analyses depending on cell composition. Another specific feature of mammary gland tissue is the low complexity of its transcriptome, because milk and whey protein genes are highly expressed, in consequence masking the expression of lowly expressed transcripts^3,4^.

To overcome these challenges, alternative approaches have been developed. Previous studies have shown that experiments on primary bovine mammary epithelial cells (pbMECs) enable the study of mammary gland functions^5–7^, but pbMECs are difficult to collect and often require killing the animal to obtain appropriate amounts of tissue. Furthermore, primary cell lines are heterogeneous, and transcriptional variation is substantially impacted by the genetic background. Moreover, after culture in *in-vitro* systems for extended time periods cells lose their original cell-type specific properties^8^, reducing the timeframe for experiments.

Milk-purified MECs can be a valuable, non-invasive source of mammary transcripts^9,10^. Furthermore, isolation of MECs from milk allows for repetitive sampling of the same animal across several time points, e.g., across the entire lactation. This enables an analysis of transcriptional regulation dynamics. In addition, cells in milk might represent cells in a distinct physiological status, whereas cells of different differentiation and functional stages are present in mammary tissue^9^.

Bovine milk contains about 10^4^ to 10^7^ cells/ml, although the variation is large between individuals, environmental conditions and time points^11,12^. Milk cell number is usually reported as number of somatic cells, which comprises the sum across a mixture of different non-epithelial cells (leukocytes)^13^. In contrast, MECs that are exfoliated from the epithelium during lactation are rare in milk and are therefore often not counted separately from somatic cells. While on average around 2% of the total milk cells constitute of epithelial cells, the proportion of MECs within the total milk cell population varies from one sample to another^14^. Since the concentration of MECs in milk is low, it is essential to collect sufficiently large volumes of milk to obtain a sufficient RNA quantity for mammary transcript analyses^15^. This limits the number of samples processed at a single collection time point, and the number of analyses that can be performed on the same sample. Nevertheless, milk cell samples can be used to monitor the mammary response to an invading pathogen. However, it is difficult to distinguish, if a change in bulk milk cell transcriptome is based on a change in cell composition or on a change in the expression of particular genes in a specific cell type. The same is true for the analysis of bulk mammary gland tissue.

Recent technical advances allow transcripts from thousands of cells to be pooled, sequenced, and subsequently identified in a single experiment at single-cell resolution^16^. This approach enables to assess the functional heterogeneity in a cell sample by identifying and characterising subpopulations of cells in a complex cell population^17–19^.

In spite of this progress in technology, the current understanding of the spectrum of molecular heterogeneity in milk cells as well as in pbMECs is still limited. In this study, we assessed the gene expression across 7,119 single-cells that were isolated from a bovine milk sample and 10,549 pbMECs using single-cell RNA sequencing (scRNA-seq). The cells were processed using the 10x Chromium Single-cell 3′ workflow. Single-cell libraries were sequenced on an Illumina HiSeq 2500 platform. Subsequent data analysis was performed with Seurat, a scRNA-seq analysis tool^20^.

The datasets presented here provide the first single-cell profiles of pbMECs and cells isolated from bovine milk. They provide a suitable reference and basis for future single-cell gene expression studies, e.g., to investigate the response of specific cells to environmental disruptors including pathogen challenges.

## Methods

### Isolation of mononuclear cells from milk

**Fig. 1:**
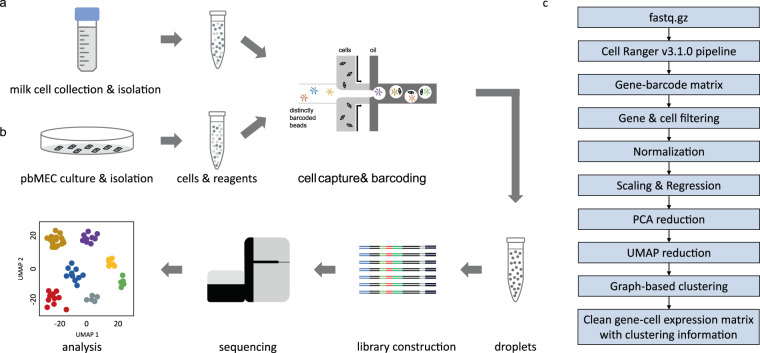
Outline of laboratory and bioinformatic workflow. The workflow comprises isolation of (**a**) mononuclear cells from milk or (**b**) pbMECs from bulk mammary gland tissue, preparation of single-cell suspensions, library construction, sequencing and (**c**) the final bioinformatic scRNA-seq workflow. Adapted from Liu *et al*.^31^.

Residual milk was collected in four 50 ml tubes from one udder quarter (total amount = 200 ml) of a clinically healthy cow immediately subsequent to a regular machine milking. The individual was in its 56^th^ week of the first lactation and had an average milk somatic cell count (SCC) of roughly 182,000 cells/ml across the previous three lactation weeks (Fig. 1a). The collection tubes were centrifuged at 3000 × g for 10 min. Subsequently, the fat layer and the supernatant were removed and 20 ml 1X PBS (Biochrom, Germany) was added to the pellet. The pellet was resuspended using wide-bore tips. After a further centrifugation for 10 min at 3000 × g, the supernatant was discarded and the pellet was resuspended in 10 ml 1X PBS. After a third centrifugation of the tube at 3000 × g for 10 min, the supernatant was discarded again, and the pellet was resuspended in 1 ml 1X PBS. The resulting cell suspension was transferred to a 2 ml low-bind DNA tube using a Flowmi cell strainer (Sigma Aldrich, USA) to remove cell debris. Then, all cells were carefully layered over 8 ml of cold Biocoll (Biochrom, Germany) with a density of 1.077 g/ml in a 15 ml tube followed by a centrifugation step at 800 × g for 30 min with the centrifuge brake set to off-mode. After centrifugation, the middle layer of the gradient was carefully collected and transferred to a 2 ml low-bind DNA tube before single-cell libraries were prepared.

### Isolation of pbMECs

Primary bovine mammary epithelial cells (pbMECs) were isolated from a healthy mammary gland quarter of a cow in the 5^th^ week of its first lactation according to the methods described by Cifrian *et al*.^21^ and Hensen *et al*.^22^ and modified as described by Yang *et al*.^23^ Initially, the skin of an udder from a freshly slaughtered cow was rinsed with 70% ethanol and subsequently removed. From the mammary parenchyma, cubes predominantly containing milk ducts and to a lesser extent secretory tissue were collected in HBSS (Hank’s Balanced Salt Solution, buffered with HEPES, supplemented with APS solution; Sigma). Minced pieces comprising preferentially epithelial areas were immersed into 30 ml HBSS and shaken continuously for five minutes. Thereafter, large clumps were allowed to sediment until the supernatant was clear of visible clumps. The sediment was homogenized in 20 ml of HBSS supplemented with 200 U/ml collagenase, Type IV. The homogenate was shaken at 37 °C and filtered through a mesh steel grid gradient (500 µm, 300 µm, 150 µm and 90 µm) (Sigma) for retrieving isolated cells. This procedure was repeated four times in 45 min intervals. Cells were collected after centrifugation (1000 × g for 10 min), washed five times in HBSS and finally plated on collagen-coated plates (Greiner Bio-One, Germany). They were cultured in RPMI1640 medium (Biochrom AG), supplemented with prolactin, hydrocortisone, insulin and 10% FCS as described previously^23^. After ~four days of unperturbed growth, fibroblasts were removed by selective trypsinization (Trypsin-EDTA (0.25%/0.02%), Biochrom AG)^23^. Pure pbMECs were cultured for further six days before harvesting and preparation of a single-cell solution (Fig. 1b).

### Concentration and viability of milk cells and pbMECs

A parallel test on inclusion of acridine orange (AO), but resistance to propidium iodide (PI) influx discriminated viable cells from dead cells, cell debris or milk micelles^24^. Briefly, the cell suspension was mixed with AO/PI (Nexcelom Bioscience Ltd, UK) in a 1:1 ratio and incubated at room temperature for five minutes. Stained objects were examined using the cell counter Auto2000 (Nexcelom, UK). Only viable cells fluoresced green, while nonviable cells fluoresced bright red and milk micelles yielded no signal. The cell counter Auto2000 was also used to determine cell concentration.

### Preparation of single-cell libraries, sequencing and alignment

Cells were prepared according to the 10x Genomics Single-cell Protocols Cell Preparation Guide (10x Genomics; CG00053, Rev C). The protocol is optimized to provide droplets containing only a single viable cell without contamination by cell-free nucleic acids or potential inhibitors of subsequent enzymatic reactions (e.g. reverse transcription). Briefly, cells were pelleted by gentle centrifugation and repeatedly washed with 1X PBS containing 0.04% BSA (400 µg/ml) and finally adjusted to a target cell concentration of 1,000 cells/µl for downstream experiments.

Single-cell RNA sequencing (scRNA-seq) libraries from pbMECs and mononuclear milk cells were generated with the Chromium Next GEM Single-cell 3′ v3.1 assay (10x Genomics) according to manufacturer’s instructions (10x Genomics User Guide Chromium Next GEM Single-cell 3′ Reagent Kits v3.1 (CG000204, Rev B)). Briefly, samples were further diluted to a concentration equivalent to a target cell recovery of 10,000 cells after sequencing and subsequently they were loaded onto a 10x Genomics Single-cell 3′ Chip together with the reverse transcription enzyme master mix.

On the 10x Genomics Single-cell 3′ Chip, cells and gel beads, which were coated with oligonucleotides to enable mRNA capture and barcoding, were partitioned into Gel Beads-in-Emulsions (GEMs). Within GEMs reverse transcription took place. The resulting cDNA for each sample was amplified and used for library preparation using the Single-cell 3′ Reagent Kit. The resulting cDNA sequencing libraries were assessed for quality and DNA concentration using a High Sensitivity DNA chip on a BioAnalyzer 2100 (Agilent Technologies) before they were sequenced on the Illumina HiSeq 2500 platform (Illumina) with sequencing parameters as recommended by 10x Genomics using two lanes per sample.

Sequencing reads were analysed using the Cell Ranger v3.1.0 alignment software provided by 10x Genomics (https://support.10xgenomics.com). Briefly, FASTQ files were obtained from HiSeq 2500 raw base call files via the Cell Ranger mkfastq pipeline. Subsequently, the FASTQ files were aligned to the *Bos taurus* ARS-UCD1.2 genome^25^ with the Ensembl 98 annotation release using STAR^26^ implemented in the Cell Ranger count pipeline, which also conducts the subsequent filtering and counting of cell barcodes and Unique Molecular Identifiers (UMIs). Those reads generated by barcode-associated cells, which passed the pipeline-internal QC, were quantified and used for establishing a gene-barcode matrix.

### Bioinformatic analysis

For data analysis, we followed best-practice recommendation for scRNA-seq analysis^27–29^ including quality control, normalization and scaling of the data, regressing out technical and unwanted biological effects, feature selection, dimensionality reduction, clustering and visualization of the data (Fig. 1c) using the scRNA-seq package Seurat v3.1.4^20^ in the R environment (R version 3.6.3). This workflow with adaptation of filtering thresholds is widely used for scRNA-seq analysis^30–34^ and described in detail below.

#### QC filtering

For data processing, we imported the gene-barcode matrices established by Cell Ranger into the Seurat environment. We filtered genes that were observed in less than 10 cells to remove genes that might originate from random noise. Regarding cells, thresholds for filtering were <300 genes expressed and <500 UMIs counted. Additionally, we removed cells with mitochondrial reads comprising more than 20% of all reads and with an overall complexity of gene expression under 0.8. After filtering, we retained 7,119 milk cells and 10,549 pbMECs, respectively (Table 1).

**Table 1:** Overview of the mapping parameters for the 10x Genomics scRNA-seq datasets established for milk cells and pbMECs.

Sample	Estimated number of cells	Number of reads	Reads mapped to genome	Mean reads per cell	Total genes detected	Median genes per cell	Number of cells post filtering
Milk cells	8,218	444,840,327	93.3%	54,130	17,953	829	7,119
pbMECs	15,486	394,325,753	91.2%	25,463	17,971	1,614	10,549

Estimates were generated by Cell Ranger on the raw data. Number of cells after QC filtering in Seurat are given in the last column.

#### Normalization, data scaling & regression

The expression data was normalized using the “NormalizeData” function of the Seurat package to reduce possible technical bias caused by differences in sequencing depths between cells^35^. The UMI count data for each gene were first divided by the total UMI counts of each cell, subsequently multiplied by the scale factor and finally log-transformed for normalization. A list of cell cycle-specific marker genes^36^ (Supplementary Table S1) served for inferring each cell’s cell cycle phase based on the cell’s respective expression levels. The function “CellCycleScoring” in the Seurat package classified the cell regarding cell cycle stage (G1, G2/M or S) and assigned respective scores to each cell. Potential sources of unspecific variation in the data were removed by regressing out the mitochondrial gene proportion, the cell cycle effect and UMI count using linear models and finally by scaling and centering the residuals as implemented in the function “ScaleData” of the Seurat package.

#### Dimensional reduction, Uniform Manifold Approximation and Projection (UMAP) & clustering

Based on expression, we identified 2,000 highly variable genes (via the function “FindVariableFeatures”). The scaled and normalized expression data of respective genes served as input for a principal component (PC) analysis, and the first 20 PCs were used to plot the variability between cells in a two-dimensional diagram by means of the UMAP procedure to reduce dimensionality of the input data. Cells were clustered into subpopulations according to the same PCs using the Seurat function “FindClusters” (resolution = 0.8), which is a graph-based clustering approach^20^.

#### Identification of cluster marker genes & cell type assignment

The function “FindAllMarkers” of the Seurat package identified genes differentially expressed between a distinct cell cluster and the other clusters in the respective dataset (milk cells, pMECs). The most differentially expressed genes can be considered cluster-specific marker genes. Cell types were assigned manually to clusters based on cell type marker expression specific to each cluster. Cell type markers were retrieved from the literature^34,37–47^ and a single-cell database^48^. Clusters with cells that expressed marker genes for a specific cell type at the highest levels were assigned the corresponding cell type label.

## Data Records

The sequencing data have been deposited in the “Functional Annotation of Animal Genomes” (FAANG) data coordination center database (https://data.faang.org/home) with accession PRJEB41576 (https://data.faang.org/dataset/PRJEB41576), which directly channels all information to the European Nucleotide Archive (ENA)^49^. A Seurat object containing the analysed data is available via Figshare for milk cells and pbMECs respectively^50^.

## Technical Validation

**Fig. 2:**
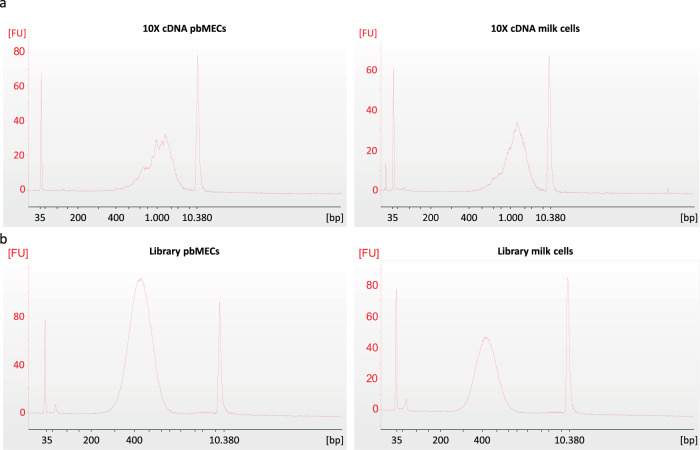
Quality control of cDNA amplification and library preparation. Concentration and quality were assessed on a BioAnalyzer with a High Sensitivity DNA Chip. Size distribution was evaluated by a standard size ladder. (**a**) Size distribution of amplified cDNA from pbMECs (left) and milk cells (right). (**b**) Fragment size distribution of the sequencing libraries obtained from pbMECs (left) and milk cells (right).

Mononuclear cells were isolated from milk, and pbMECs were harvested after primary cell isolation and initial cell culture. GEMs were prepared with a Chromium Controller (10x Genomics, Pleasanton, CA). Following initial cDNA synthesis, the output was amplified with 13 or 11 PCR cycles for milk cells and pbMECs, respectively. Quality control as well as quantification of the resulting PCR products was determined with a DNA high sensitivity assay on a BioAnalyzer 2100 (Agilent Technologies). The peak of the fragment size distribution was around 1,300 to 1,500 bp (Fig. 2a) and therefore indicated a good quality of cDNA synthesis. After cDNA fragmentation, adaptor ligation and PCR amplification, the resulting library quality was evaluated via a BioAnalyzer 2100 (Agilent Technologies) (Fig. 2b).

After sequencing of libraries on two lanes per sample on an Illumina HiSeq 2500 and processing of raw sequencing data with Cell Ranger v3.1.0, reads were analysed with Seurat^20^. Both milk cell and pbMEC libraries achieved a high mapping rate for reads of 93.3% and 91.2%, respectively (Table 1), and the mapping metrics showed a similar pattern between the samples (Supplementary Table S2). For an average of 83.9% and 82.7% bases of milk cell and pbMEC reads, respectively, the phred quality score was 30 and beyond (Q30), indicating that high-quality mapping data were generated for downstream analyses^51^. We detected a median number of 829 expressed genes per cell for the milk cells and a median of 1,614 expressed genes per cell for the pbMECs.

Because it is essential that feature count data are obtained from viable cells, we applied strict data thresholds to exclude data that could originate from damaged cells or other technical issues^52^. A high percentage of mitochondrial transcripts indicates cells in apoptosis^52,53^, presumably due to the loss of cytoplasmic RNA from perforated cells leading to a relative enrichment of mitochondrial transcripts. There is a particular risk for apoptotic cells within the MECs due to a cell turnover of the secretory tissue^54^. Hence, we computed the mitochondrial transcript to gene read ratio in both samples and removed cells with a ratio of >0.2. We also removed cells with <500 distinct reads in a cell and <300 expressed genes detected per cell, as these indicate damaged cells. Additionally, we calculated the library complexity and filtered the data for a complexity >0.8. Supplementary Figure S1 illustrates the number of genes, unique molecular identifiers (UMIs), the percentage of mitochondrial transcript reads in each cell and the library complexity of the milk cell (Supplementary Figure S1a) and the pbMEC (Supplementary Figure S1b) dataset before and after applying the filter criteria. After QC filtering, we retained high quality data for 7,119 cells isolated from milk and 10,549 pbMECs.

Filtered raw read counts were normalized in order to account for differences in sequencing depth per cell. However, normalized data may still contain biological noise such as mitochondrial gene expression or effects of the cell cycle phase causing variability beyond the study design, which should be excluded from the data^55^. Therefore, we assigned each cell a cell cycle score based on the expression of the cell cycle gene markers. To remove all signals related to cell cycle, quantitative scores for the cell cycle phases were used in downstream analyses. According to the cell cycle scores, 3,743 pbMECs and 1,195 milk cells were assigned to the G2M phase, 3,264 pbMECs and 2,146 milk cells to the S phase and 3,542 pbMECs and 3,778 milk cells to the G1 phase. All clusters of the pbMECs were equally represented in the pbMECs data cell cycle scores, whereas the distribution of the cell cycle scores varied in the milk cell dataset clusters (Supplementary Figure S2). In addition, we scaled the data and regressed against gene count and mitochondrial gene expression.

**Fig. 3:**
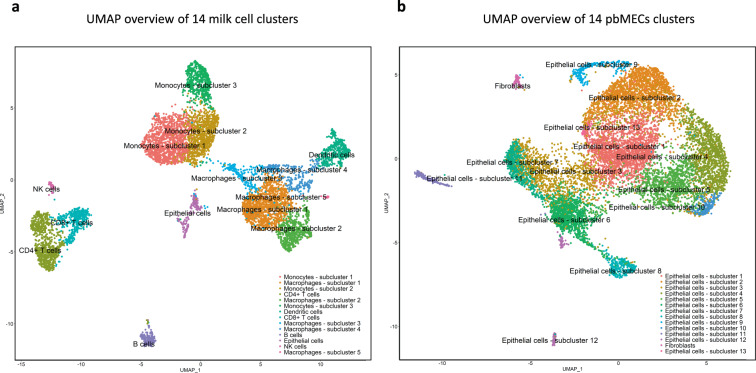
Clustering of single cell data in milk and pbMECs. (**a**) Two dimensional cell clustering via Uniform Manifold Approximation and Projection (UMAP) from the first 20 principal components for the population of 7,119 milk cells. Cluster allocation of each cell is indicated by color. (**b**) Two dimensional cell clustering via Uniform Manifold Approximation and Projection (UMAP) from the first 20 principal components for the population of 10,549 pbMECs. Cluster allocation of each cell is indicated by color.

We used the scaled datasets to identify variable expressed genes and performed a dimensional reduction via PC analysis, and visualized the data using UMAP (Fig. 3). The pbMECs formed 14 indistinct clusters, whereas the milk cells formed 14 separated clusters. Differential expressed genes between clusters were identified. The 10 top-scoring genes per cluster can be found in Supplementary Table S3 for milk cell clusters and Supplementary Table S4 for pbMEC clusters, respectively. Cell cycle phase and cluster assignment are reported in Data Table 1 and Data Table 2 for the milk cell and pbMEC dataset, and can be accessed via Figshare^50^.

**Fig. 4:**
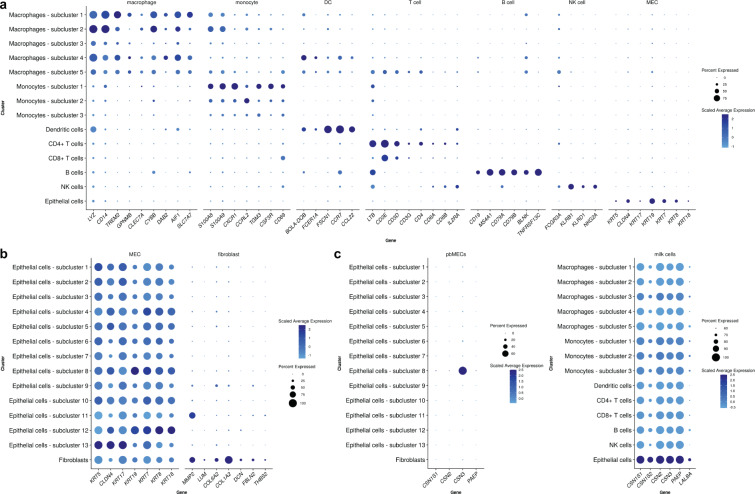
Analysis of cell type specific marker gene expression. The dot size encodes the proportion of cells that express the gene, while the color encodes the scaled average expression level across those cells (dark blue is high). At the top of each dot plot, the cell types, in which the marker genes are expressed (**a**,**b**) or the datasets (**c**) are indicated (**a**) Expression of immune cell (macrophage, monocyte, DC, T cell, B cell, NK cell) and epithelial cell marker genes (MEC) in the clusters of the milk cell dataset. (**b**) Expression of epithelial cell (MEC) and fibroblast (fibroblast) marker genes in the pbMEC clusters. Epithelial cell markers are expressed in all clusters (left), whereas the expression of fibroblast markers is restricted to one cluster (right). (**c**) Expression of casein (*CSN1S1*, *CSN1S2*, *CSN2*, *CSN3*) and whey protein (*LALBA*, *PAEP*) genes in pbMECs (left) and milk cells (right). *CSN1S2* and *LALBA* were not expressed in pbMECs.

From the literature^12,14,56^, we expected to find several immune cell types including monocytes, macrophages and lymphocytes (T cells and B cells) as well as a low number of MECs in the milk cell dataset. Accordingly, we looked at the expression of immune cell^34,37–42,48^ and MEC markers^43,44^ throughout the clusters of this dataset for validation that the established dataset is indeed representative of a milk cell population (Fig. 4a). We detected that the cluster, which comprises 2.5% cells (n = 178) of the dataset was the only cluster, in which cells clearly expressed B cell markers, whereas the cluster, which contains 2.47% of all cells (n = 176), expressed epithelial cell markers (*KRT5*, *KRT7*, *KRT8*, *KRT17*, *KRT18*, *KRT19* and *CLDN4*, Fig. 4a). Two cell clusters contain cells dominantly expressing T cell markers, whereas cells of another cluster expressed NK cell markers. The expression patterns of three clusters were very similar. These clusters mainly expressed monocyte markers. Dendritic cell markers were expressed by a cluster containing 446 cells. Lymphocytes and macrophages are the dominant cell types in milk of healthy mammary glands^14,56^ and form numerous different sub-populations with distinct functions. Distribution of the milk cells across clusters is reported in Supplementary Table S3.

For the pbMEC dataset, we expected the cells to express characteristic epithelial cell markers, i.e., cytokeratins and claudins^43,44^. All pbMECs clusters abundantly expressed *KRT5*, *KRT7*, *KRT8*, *KRT17*, *KRT18*, *KRT19* and *CLDN4* (Fig. 4b). Furthermore, eight of the 14 clusters displayed cytokeratin genes within the top 10 cluster-specific marker genes (Supplementary Table S4).

Isolation of the pbMECs was performed from bulk mammary tissue with a trypsinization step to exclude fibroblasts. Thus, it was likely that the investigated pbMEC population still contained some fibroblasts. Hence, we checked the expression of fibroblast markers, i.e., *MMP2*, *LUM*, *COL6A2*, *COL1A2*, *DCN*, *FBLN2* and *THBS2*^45–47^. Indeed, all these genes were mainly expressed in one cluster (Fig. 4b). Therefore, we could conclude that this cluster of the pbMEC dataset is composed of fibroblasts (0.69% of the cells in the dataset (n = 73)). However, taken together, the pbMEC clusters are fairly homogeneous. Distribution of the pbMECs across clusters is reported in Supplementary Table S4. From the results of cluster expression profiles, we can conclude that the cell populations studied display characteristic expression profiles for pbMECs and bovine milk cells, respectively.

Additionally, we checked the expression of casein and whey protein genes^57^ throughout both datasets (Fig. 4c). The mammary tissue and milk cells of lactating dairy is known for a low-complexity transcriptome with milk protein genes (caseins, whey proteins) as the most abundant transcripts accounting for up to 70% of all transcripts expressed^4,58^. Indeed, milk protein genes were expressed in all clusters of the milk cell dataset, with the highest expression level in the cluster, which contains cells expressing epithelial cell markers and most likely represent MECs. On the other hand, only a few cells in the pbMEC dataset expressed casein and whey protein genes, with epithelial cells in subcluster 8 showing the highest expression level. In the lactating mammary gland, two different types of epithelial cells can be discriminated: myoepithelial and luminal epithelial cells^59^. Luminal epithelial cells comprise ductal epithelial cells lining the ducts of the mammary gland, and alveolar epithelial cells, which are the actual secretory cells in the mammary gland^60^. The expression pattern of pbMECs in our study clearly reflects the sampling protocol of the cells, which have been mainly isolated from ductal regions of udder tissue. Our data on cell type distribution in the pbMEC dataset are in line with this sampling preference, because the proportion of ductal epithelial cells, which do not secrete milk proteins and therefore do not express the respective genes, seems to be considerably higher than the proportion of cells with milk protein expression representing presumably alveolar cells.

Taken together, our datasets represent a valuable resource for dissecting cell-to-cell gene expression variation and cell population heterogeneity of bovine mononuclear milk cells and, to a lesser extent, pbMECs. Additionally, these data could be a reference dataset for bovine milk cell analyses, as the dataset displays the different milk cell populations varying in ontogenetic and original background.

## Supplementary information

Supplementary Material

## Data Availability

Auxiliary File 1 (milk cells) and Auxiliary File 2 (pbMECs) contain the R code (R version 3.6.3) for scRNA-seq data processing and subsequent visualization of the output. They are publicly accessible via Figshare^50^.
