# Supplementary material for: Single-cell RNA sequencing of freshly isolated bovine milk cells and cultured primary mammary epithelial cells
Source: Sci Data. 2021 Jul 15;8:177. doi: 10.1038/s41597-021-00972-1 (PMC8282601; doi:10.1038/s41597-021-00972-1)
Supplement: Supplementary file 1 — Supplementary Material [file 41597_2021_972_MOESM1_ESM.pdf]

Supplementary material for "Single-cell RNA sequencing of freshly isolated bovine milk cells and cultured primary mammary epithelial cells"

Authors

Doreen Becker[1], Rosemarie Weikard[1], Frieder Hadlich[1], Christa Kühn[1,2]

Affiliations

1. Leibniz Institute of Farm Animal Biology (FBN), Institute of Genome Biology, Wilhelm-Stahl-Allee 2, 18196 Dummerstorf, Germany
2. University of Rostock, Faculty of Agricultural and Environmental Sciences, Justus-von-Liebig-Weg 6, 18059 Rostock, Germany

Corresponding author: Doreen Becker (becker.doreen@fbn-dummerstorf.de)

List of Tables

Table S1. Specific cell cycle phase genes……2
Table S2. Mapping metrics of milk cells and pbMECs……3
Table S3. Top 10 cluster marker genes milk cells……4
Table S4. Top 10 cluster marker genes pbMECs……5

List of Figures

Figure S1. Visualization of QC metrics before and after QC filtering……6
Figure S2. Cell cycle phase distribution throughout the pbMEC and milk cell clusters……7

Table S1. Specific cell cycle phase genes

cell cycle stage	geneID	gene name	cell cycle stage	geneID	gene name
S	ENSBTAG00000051782	*ATAD2*	G2/M	ENSBTAG00000009218	*ANLN*
S	ENSBTAG00000020301	*BLM*	G2/M	ENSBTAG00000016730	*ANP32E*
S	ENSBTAG00000012068	*BRIP1*	G2/M	ENSBTAG00000013009	*AURKA*
S	ENSBTAG00000011313	*CASP8AP2*	G2/M	ENSBTAG00000001717	*AURKB*
S	ENSBTAG00000004906	*CCNE2*	G2/M	ENSBTAG00000013573	*BIRC5*
S	ENSBTAG00000004286	*CDC45*	G2/M	ENSBTAG00000021181	*BUB1*
S	ENSBTAG00000010384	*CDC6*	G2/M	ENSBTAG00000006246	*CBX5*
S	ENSBTAG00000003458	*CDCA7*	G2/M	ENSBTAG00000005269	*CCNB2*
S	ENSBTAG00000018864	*CENPU*	G2/M	ENSBTAG00000009819	*CDC20*
S	ENSBTAG00000011880	*CHAF1B*	G2/M	ENSBTAG00000005293	*CDC25C*
S	ENSBTAG00000002826	*CLSPN*	G2/M	ENSBTAG00000002756	*CDCA2*
S	ENSBTAG00000015338	*DSCC1*	G2/M	ENSBTAG00000019777	*CDCA3*
S	ENSBTAG00000018142	*DTL*	G2/M	ENSBTAG00000014326	*CDCA8*
S	ENSBTAG00000017446	*E2F8*	G2/M	ENSBTAG00000010109	*CDK1*
S	ENSBTAG00000009396	*EXO1*	G2/M	ENSBTAG00000023938	*CENPA*
S	ENSBTAG00000000064	*FEN1*	G2/M	ENSBTAG00000009035	*CENPE*
S	ENSBTAG00000044006	*GINS2*	G2/M	ENSBTAG00000024449	*CENPF*
S	ENSBTAG00000017329	*GMNN*	G2/M	ENSBTAG00000021162	*CKAP2*
S	ENSBTAG00000005979	*HELLS*	G2/M	ENSBTAG00000002298	*CKAP2L*
S	ENSBTAG00000014380	*MCM2*	G2/M	ENSBTAG00000004448	*CKAP5*
S	ENSBTAG00000017021	*MCM4*	G2/M	ENSBTAG00000024476	*CKS1B*
S	ENSBTAG00000015595	*MCM5*	G2/M	ENSBTAG00000001938	*CKS2*
S	ENSBTAG00000015172	*MCM6*	G2/M	ENSBTAG00000013757	*CTCF*
S	ENSBTAG00000002742	*MSH2*	G2/M	ENSBTAG00000002331	*DLGAP5*
S	ENSBTAG00000015346	*NASP*	G2/M	ENSBTAG00000023814	*ECT2*
S	ENSBTAG00000006065	*PCNA*	G2/M	ENSBTAG00000001617	*G2E3*
S	ENSBTAG00000008170	*POLA1*	G2/M	ENSBTAG00000049308	*GAS2L3*
S	ENSBTAG00000016869	*POLD3*	G2/M	ENSBTAG00000007102	*GTSE1*
S	ENSBTAG00000044000	*PRIM1*	G2/M	ENSBTAG00000024726	*HJURP*
S	ENSBTAG00000002918	*RAD51*	G2/M	ENSBTAG00000015101	*HMGB2*
S	ENSBTAG00000040065	*RAD51AP1*	G2/M	ENSBTAG00000014773	*HMMR*
S	ENSBTAG00000018589	*RFC2*	G2/M	ENSBTAG00000030170	*JPT1*
S	ENSBTAG00000006225	*RPA2*	G2/M	ENSBTAG00000009383	*KIF11*
S	ENSBTAG00000013111	*RRM1*	G2/M	ENSBTAG00000005708	*KIF20B*
S	ENSBTAG00000008216	*RRM2*	G2/M	ENSBTAG00000009983	*KIF23*
S	ENSBTAG00000001328	*SLBP*	G2/M	ENSBTAG00000015280	*KIF2C*
S	ENSBTAG00000000372	*TIPIN*	G2/M	ENSBTAG00000008453	*LBR*
S	ENSBTAG00000007003	*TYMS*	G2/M	ENSBTAG00000002444	*MKI67*
S	ENSBTAG00000004310	*UBR7*	G2/M	ENSBTAG00000014730	*NCAPD2*
S	ENSBTAG00000002224	*UHRF1*	G2/M	ENSBTAG00000021673	*NDC80*
S	ENSBTAG00000020451	*USP1*	G2/M	ENSBTAG00000009618	*NEK2*
S	ENSBTAG00000017215	*WDR76*	G2/M	ENSBTAG00000007247	*NUF2*
			G2/M	ENSBTAG00000010774	*NUSAP1*
			G2/M	ENSBTAG00000002981	*PIMREG*
			G2/M	ENSBTAG00000018806	*PSRC1*
			G2/M	ENSBTAG00000012481	*RANGAP1*
			G2/M	ENSBTAG00000005862	*SMC4*
			G2/M	ENSBTAG00000011044	*TACC3*
			G2/M	ENSBTAG00000004240	*TMPO*
			G2/M	ENSBTAG00000019262	*TOP2A*
			G2/M	ENSBTAG00000018775	*TPX2*
			G2/M	ENSBTAG00000005456	*TTK*
			G2/M	ENSBTAG00000052518	*TUBB4B*
			G2/M	ENSBTAG00000016746	*UBE2C*

Table S2. Mapping metrics of milk cells and pbMECs

Sample	Reads mapped to Genome	Reads mapped confidently to genome	Q30 Bases in RNA Read	Reads mapped confidently to intergenic regions	Reads mapped cofidently to intronic regions	Reads mapped confidently to exonic regions	Reads mapped confidently to transcriptome	Reads mapped antisense to gene
**milk cells**	93.9%	88.3%	83.9%	11.9%	16.3%	60.0%	57.2%	0.9%
**pbMECs**	91.2%	85.4%	82.7%	10.2%	11.4%	63.9%	61.0%	0.8%

Table S3. Top 10 cluster marker genes milk cells

Cluster	Number of cells	Inferred cell type	Top 10 marker genes
0	1,296	Monocytes - subcluster 1	*TGM3,S100A9, CXCR1, ENSBTAG00000048980, CSF3R, IL1B, S100A8, CD69, PLAUR, ISG15*
1	1,200	Macrophages - subcluster 1	*LGMN, LGALS3, CSTB, CTSS, CD9, LIPA, CTSB, CCL2, APOE, SPP1*
2	904	Monocytes - subcluster 2	*CCL3, CCRL2, CSF1, CXCR4, CCL4, ENSBTAG00000004714, ICAM1, CCL5, DEFB10, NUPR1*
3	768	CD4+ T cells	*CD52, UBD, GIMAP7, ENSBTAG00000027204, GIMAP7.2, CD3E, ENSBTAG00000034609, ENSBTAG00000037510, GIMAP7.3, GIMAP7.1*
4	585	Macrophages - subcluster 2	*LYZ, DEFB13, DEFB7, VCAN, FCN1, CD14, ARAF, S100A4, CCL2, CXCL5*
5	557	Monocytes - subcluster 3	*TUBB4B, DNAJB1, HSPA8, UBB, GADD45A, HSPA1A, HSP90AA1, GADD45G, HSPH1, ID2*
6	446	Dendritic cells	*CCR7, CCL22, LY75, FSCN1, TBC1D4, CCDC88A, PPP1R14A, CRIP1, LSP1, BLA-DQB*
7	395	CD8+ cells	*CTSW, ENSBTAG00000000144, SPRY2, ENSBTAG00000009943, KIT, PIK3R1, FAM162A, ENSBTAG00000055197, RGS1, SOD1*
8	263	Macrophages - subcluster 3	*CSTB.1, LGALS3, FABP5, FTL, FTH1, PRDX1, CSTB, C11H9orf16, ANXA5, ATOX1*
9	261	Macrophages - subcluster 4	*IFI30, ENSBTAG00000009656, ENSBTAG00000037605, CD74, BOLA-DQB, ENSBTAG00000013919, BLA-DQB, BOLA-DRA, CST3, APOE*
10	178	B cells	*ENSBTAG00000047700, ENSBTAG00000055240, MS4A1, TNFRSF13C, CD79A, BANK1, PHGDH, BOLA-DRA, MTPN, ENSBTAG00000048781*
11	176	Epithelial cells	*KRT19, CLDN4, LTF, WFDC2, CRYAB, CSN3, SCD, PAEP, CSN1S1, ENSBTAG00000052369*
12	68	NK cells	*CRABP1, TMIGD2, ENSBTAG00000009943, S100A13, CTSW, HOPX, GPR171, ENSBTAG00000037510, GIMAP7.3, GIMAP4*
13	22	Macrophages - subcluster 5	*MKI67, PCLAF, TOP2A, CENPF, TPX2, STMN1, DUT, HMGB1, HMGB2, H2AFV*

Table S4. Top 10 cluster marker genes pbMECs

Cluster	Number of cells	Inferred cell type	Top 10 marker genes
0	2,095	Epithelial cells - subcluster 1	*ENSBTAG00000051082, COL17A1, FAT1, LAMB3, F3, TGFBI, ITGA6, DSP, GJB2, DUSP1*
1	1,694	Epithelial cells - subcluster 2	*SFN, RPL12, PDLIM1, S100A16, RPS3, RPLP0, RHOD, RPS12, RPLP1, KRT14*
2	1,508	Epithelial cells - subcluster 3	*ND2, COX3, ATP6, CYTB, ENSBTAG00000043570, ND4, ND1, ENSBTAG00000043567, ND3, TAF1D*
3	1,217	Epithelial cells - subcluster 4	*MUSTN1, MIA, KRT7, SH3BGRL3, CRYAB, WFDC2, SPP1, KRT18, KRT15, FGFBP1*
4	1,214	Epithelial cells - subcluster 5	*THBS1, PSAP, AHNAK, SPP1, SPTBN1, MAL2, UTRN, MAP1B, FN1, KRT15*
5	1,121	Epithelial cells - subcluster 6	*TXN, ENSBTAG00000052579, WFDC2, LGALS1, KRT19, ATP5F1E, CRYAB, CD24, CST6, ENSBTAG00000049572*
6	625	Epithelial cells - subcluster 7	*ENSBTAG00000043567, ENSBTAG00000043570, COX2, COX3, ATP6, ND4, ND3, ND1, ND2, ND4L*
7	264	Epithelial cells - subcluster 8	*APOA1, LTF, CSN3, KRT19, PLAT, CALCB, NES, WFDC2, ENSBTAG00000049572, CST6*
8	242	Epithelial cells - subcluster 9	*SMOC1, SFRP2, COL1A1, COL5A1, CCDC80, VIM, HTRA1, CTHRC1, SERPINE1, NPPB*
9	189	Epithelial cells - subcluster 10	*ENSBTAG00000038770, SELENBP1, MT1A, PPDPF, MIA, KRT15, MT1E.1,TNFRSF6B, MT2A, SPP1*
10	156	Epithelial cells - subcluster 11	*SELP, PTX3, SELE, OLR1, GNG11, GPX3, ANKRD1, RGCC, PRSS2, PLAT*
11	107	Epithelial cells - subcluster 12	*HSD3B1, STEAP4, CCL2, CFB, CD24, KRT19, KRT18, CRCT1, WFDC2, TGM2*
12	73	Fibroblasts	*VCAN, COL3A1, COL1A2, COL1A1, MMP2, NTS, COL4A1, COL5A1, SERPINE1, TNC*
13	44	Epithelial cells - subcluster 13	*DSG1, SBSN, TGM3, KRT6B, S100A9, LYPD3, S100A8, ENSBTAG00000023026, S100A12, TM4SF1*

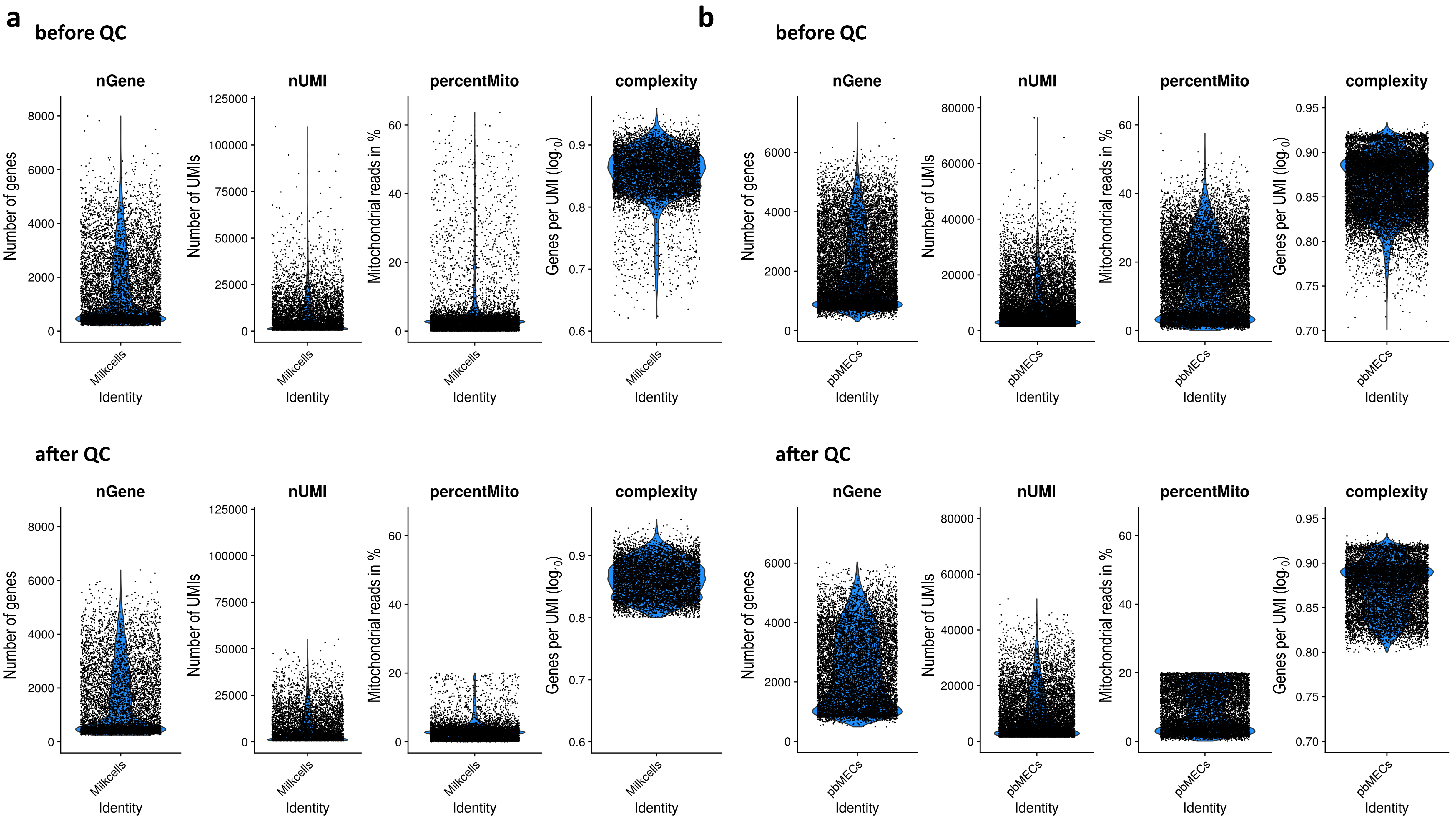

Figure S1. Visualization of QC metrics before (upper panel) and after (lower panel) QC filtering for milk cells (a) and pbMECs (b). We removed cells with < 300 expressed genes detected per cell (nGene) and < 500 distinct molecules in a cell (nUMI). We also removed cells with mitochondrial reads comprising more than 20% of all reads (percentMito). Additionally, we calculated the library complexity from the data and filtered the data for a complexity < 0.8 (complexity).

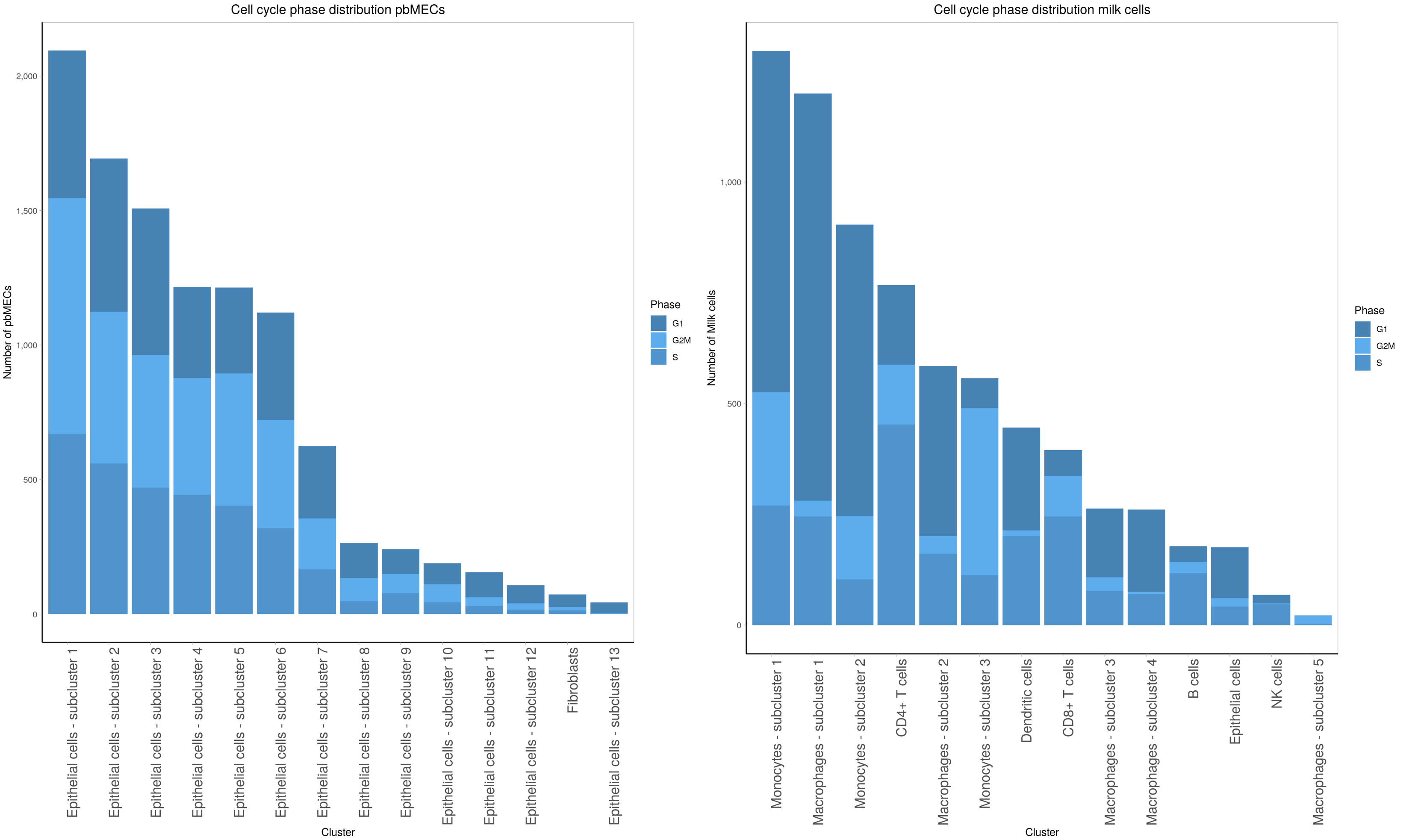

Figure S2. Cell cycle phase distribution throughout the assigned clusters of the pbMEC and milk cell data set. Distribution of the cell cycle scores was homogenous in pbMECs and varied in the milk cell data set clusters.
